# Thou Shalt Not Steal

**Published:** 1887-04

**Authors:** 


### THOU SHALT NOT STEAL.

Probably in the whole range of human experience there is no phase of existence which involves so much unalloyed happiness as that of the editor of a medical journal. So many immunities and privileges are incident to this department of human effort that the happy incumbent may be said to be raised above the plane of ordinary human existence and to dwell in a paradise of his own. Not the least of the many privileges which fall to his lot and which distinguish him from his less fortunate fellow-beings is his absolute freedom from the restraints imposed by the eighth commandment. To him the distinction between *meum* and *tuum* is a "distinction without a difference." This is an inherent and incontestable right which belongs only to the editor of a medical journal.

Our somewhat restricted range of observation has not enabled us to determine whether this independence of the laws ordinarily governing the possession of property is subject to any limitation. We infer, however, that it is not. It would be manifestly inconsistent, even absurd, to say that an editor might appropriate without acknowledgment the products of another's intellectual labor, and yet must not at the same time take his watch or his pocket-book if he should choose. We can conceive of no sophistry which would justify such a paradoxical view.

Yet, we must confess that down in the depths of our heart we have a lingering notion that the editor of a medical journal is only an ordinary mortal after all, and that he is therefore amenable to the obligations of common honesty. This idea is perhaps a mere prejudice or a result of injudicious early training and our possession of it probably proves us to be wofully behind the times. Still, we cannot help feeling that for us to steal the thoughts of a contemporary and publish them without credit would be a dishonorable and morally inexcusable act, even though we be the editor of a medical journal; and so, when we find an article, a translation or an abstract which has cost us time, labor or money, appropriated bodily and published without giving us credit, the old-fashioned notion referred to leads us to believe that such a course is a dishonest and a dishonorable one and we cannot help feeling correspondingly aggrieved thereby.

Our view of the matter obviously places us in the minority. Every week brings us journals whose editors have availed themselves of their editorial privileges in this respect at our expense. No less than half a dozen such lie before us now. Hence, we are constrained to believe, with so many examples to prove it, that the eighth commandment is not meant for the editors of medical journals. We are not yet prepared, however, to adopt this view in our own practice, so contrary is it to all our pre-conceived ideas and habits. As anxious as we are to "keep up with the procession" in all that is right and honorable, we shall for the present be content to lag behind in this regard along with the respectable minority who still cling to time-honored ideas of the ethics of honest journalism.

